# PIWI-interacting RNA (piRNA): a narrative review of its biogenesis, function, and emerging role in lung cancer

**DOI:** 10.2478/abm-2022-0002

**Published:** 2022-02-28

**Authors:** Pritha Mukherjee, Shamee Bhattacharjee, Deba Prasad Mandal

**Affiliations:** Department of Zoology, West Bengal State University, Berunanpukuria, Malikapur, Barasat, Kolkata 700126, West Bengal, India

**Keywords:** PIWI protein, Drosophila, RNA, small interfering, Argonaute proteins, lung neoplasms, epigenetic repression

## Abstract

Cancer remains elusive in many aspects, especially in its causes and control. After protein profiling, genetic screening, and mutation studies, scientists now have turned their attention to epigenetic modulation. This new arena has brought to light the world of noncoding RNA (ncRNA). Although very complicated and often confusing, ncRNA domains are now among the most attractive molecular markers for epigenetic control of cancer. Long ncRNA and microRNA (miRNA) have been studied best among the noncoding genome and huge data have accumulated regarding their inhibitory and promoting effects in cancer. Another sector of ncRNAs is the world of PIWI-interacting RNAs (piRNAs). Initially discovered with the asymmetric division of germline stem cells in the *Drosophila* ovary, piRNAs have a unique capability to associate with mammalian proteins analogous to P-element induced wimpy testis (PIWI) in *Drosophila* and are capable of silencing transposons. After a brief introduction to its discovery timelines, the present narrative review covers the biogenesis, function, and role of piRNAs in lung cancer. The effects on lung cancer are highlighted under sections of cell proliferation, stemness maintenance, metastasis, and overall survival, and the review concludes with a discussion of recent discoveries of another class of small ncRNAs, the piRNA-like RNAs (piR-Ls).

Lung cancer causes the highest mortality in cancer-related deaths worldwide [1]. One of the major causes of lung cancer is tobacco smoking, which may be associated with socioeconomic status [2, 3]. While raising awareness is necessary to control the growing incidence of lung cancer, understanding the cellular networks and discovering new targets for therapies are also important. In recent trends, scientists have examined the pool of noncoding RNAs (ncRNAs) and their specific roles in epigenetic regulation. So far, the most studied ncRNAs are microRNAs (miRNAs) and their regulatory roles in various cancers, including lung cancer, have been established [4].

P-element induced wimpy testis (PIWI) proteins belong to the Argonaute family of proteins [5], which were first discovered in *Drosophila melanogaster* ovarian germ cells and follicular cells [6]. By interacting with Tudor domain-containing proteins (TDRDs), PIWI proteins mediate the biogenesis of PIWI-interacting RNAs (piRNAs), thereby silencing transposons [7]. Three types of PIWI subfamily proteins are found in *Drosophila*—piwi, which localizes in the nucleus of germ and gonadal somatic cells, Aubergine (aub), and Archipelago 3 or Argonaute 3 (ago3), which are both expressed in nuages, or germline granules, which are special cytoplasmic compartments in *Drosophila melanogaster*. All these proteins are involved in the biogenesis of piRNAs in *Drosophila* flies [8]. PIWI proteins use piRNAs as their guide to the specific DNA sequences for transposon silencing and gene regulation as well as playing major roles in their biogenesis [9]. Orthologs of *Drosophila* piwi proteins have been found in mice, zebrafish, *C*. *elegans*, and humans, in which they have similar roles in maintaining male and female fertility (**Figure 1**). In the human, genome 4 piwi orthologs (PIWIL1/HIWI, PIWIL2/HILI, PIWIL3, and PIWIL4/HIWI2) have been identified [5]. The first evidence of human PIWI association with cancer was studied in seminomas [14]. Aberrant expression of 1 or more of these 4 proteins is common, especially in breast, prostate, and colorectal carcinomas (**Table 1**). Limited findings have emerged in the case of lung cancer [4, 60,61,62]. Various studies have found interesting patterns of human PIWI-like protein expression and regulatory effects in lung cancers [63]. Human PIWIs and piRNAs may be important biomarkers and therapeutic targets for various cancers [64, 65].

**Figure 1:**
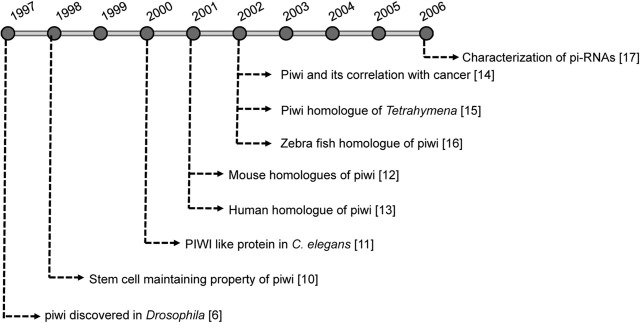
Early discoveries related to PIWI proteins and piRNA. piRNA, PIWI-interacting RNA; PIWI, P-element induced wimpy testis protein.

**Table 1:** Important discoveries of PIWI/piRNA expression in various cancers^†^

**Year**	**PIWI/piRNA**	**Expression**	**Cancer type**	**Role**
2005	PIWIL2	Upregulated	Testicular seminoma	Inhibition of apoptosis and promotion of proliferation via Stat3/Bcl-X_L_ signaling pathway [18]
2006	PIWIL1	Upregulated	Human gastric cancer	Cell proliferation [19]
2007	PIWIL1	Upregulated	Soft tissue sarcoma	Stem cell proliferation [20]
2008	PIWIL1	Up/down-regulated	Adenocarcinoma	Poor prognosis and death [21]
2009	PIWIL1	Presence in cytoplasm	Esophageal squamous cell carcinoma	Poor prognosis [22]
2010	PIWIL2	Varied	Cervical neoplasia	Biomarker [23]
2010	PIWIL2	Upregulated	Human breast cancers	Biomarker [24]
2011	PIWIL1	Upregulated	Glioma	Tumor progression, poor outcome, and biomarker [25]
2011	piR-651	Upregulated	Gastric, colon, lung, and breast cancer	Increases cell proliferation [26]
2011	PIWIL1	Upregulated	Colorectal cancer	Leads to poor overall survival, biomarker [27]
2012	piR-823	Downregulated	Gastric cancer	Increases cell proliferation [28]
2012	PIWIL2	Upregulated	Colon cancer	Metastasis [29]
2013	PIWI	Upregulated	Stage III epithelial ovarian cancer	Promotes metastasis, biomarker [30]
2013	piR-932	Upregulated	Breast cancer	Positive regulator of breast cancer stem cells [31]
2014	PIWIL1	Upregulated	Human breast cancer	Cell proliferation [32]
2014	piRNA-823	Upregulated	Multiple myeloma	Regulates angiogenesis [33]
2014	PIWIL1 and PIWIL4	Varied	Renal cell carcinoma	Related to clinicopathological parameters [34]
2014	PIWIL1	Upregulated	Cervical cancer	Promotes chemoresistance [35]
2014	PIWIL1	Upregulated	Hepatocellular carcinoma	Reduces proliferation and migration [36]
2015	piRNA-DQ594040	Downregulated	Bladder cancer	Promotes cell proliferation, colony formation and functions against apoptosis [37]
2015	piR-021285	Upregulated	Breast cancer	Epigenetic remodeling [38]
2015	PIWIL2	Upregulated	Prostate cancer	Metastasis [39]
2015	piR-017061	Downregulated	Pancreatic cancer	Associated with diseased condition [40]
2015	piR-015551	Downregulated	Colorectal cancer	Associated with long ncRNA expression [41]
2015	PIWIL1	Downregulated	Chronic myeloid leukemia	Induces growth and metastasis [42]
2015	PIWIL2	Upregulated	Cholangiocarcinoma	Involved in shorter survival span and metastasis [43]
2015	piR-57125	Downregulated	Renal cell carcinoma	Associated with tumor recurrence and metastasis, prognostic biomarker [44]
	piR-30924	Upregulated		
	piR-38756	Upregulated		
2015	PIWIL1	Upregulated	Type 1 endometrial cancer	Tumor progression by downregulating PTEN [45]
2015	PIWIL2	Downregulated	Bladder cancer	Related to disease specific and progression free survival [46]
2016	PIWIL4	Upregulated	Breast cancer	Metastasis, antiapoptotic activity and proliferation[47]
2016	FR140858	Differentially expressed	Head and neck squamous cell carcinoma	Correlated to human papillomavirus infection [48]
2016	piR-598	Upregulated	Glioma	Induces growth and proliferation [49]
2017	PIWIL3	Upregulated	Melanoma	Induces metastasis [50]
2017	PIWIL4	Upregulated	Retinoblastoma	Induces proliferation [51]
2018	piR-5937	Varied	Colon cancer	Biomarker [52]
	piR-28876			
2018	piR-8041	Downregulated	Glioblastoma	Related to tumor growth [53]
2018	PIWIL1	Upregulated	Gastric cancer	Metastasis [54]
2018	PIWIL1 and PIWIL2	Varied	Muscle invasive urothelial bladder cancer	Correlation with clinical factors, biomarkers [55]
2018	PIWIL4	Upregulated	Human breast cancer	Cell motility [56]
2019	piR-823	Upregulated	Multiple myeloma	Tumor progression [57]
2019	piR-39980	Upregulated	Neuroblastoma	Tumor progression and drug resistance [58]
2019	piR-36712	Downregulated	Breast cancer	Chemoresistance and tumor progression [59]
2020	piR-004987	Upregulated	Lung cancer	Correlated with lung cancer from sputum as compared with normal [60]
	piR-020809	Upregulated		
	piR-023338	Downregulated		
	piR-011186	Downregulated		
2021	piR-hsa-211106	Downregulated	Lung cancer	Chemoresistance and tumor progression [61]
2021	piR-1008	Upregulated	Lung cancer	
	piR-28231	Upregulated	Lung cancer	
	piR-11256	Upregulated	Lung cancer	
	piR-30636	Upregulated	Lung cancer	
	piR-24143	Upregulated	Lung cancer	Biomarker lung cancer [62]
	piR-6842	Upregulated	Lung cancer	
	piR-8757	Upregulated	Lung cancer	
	piR-15572	Upregulated	Lung cancer	
	piR-5444	Upregulated	Lung cancer and serum exosome	
	piR-26925	Upregulated		

† Lung cancer until 2021 and other cancers until 2019. PIWI, PIWI protein (human); piRNA, Piwi protein-interacting RNA; PIWIL1, PIWI-like 1 protein (human) or HIWI protein (human); PIWIL2, HILI protein (human); PIWIL3, HIWI3 protein (human); and PIWIL4, HIWI2 protein (human); ncRNA, non-coding RNA.

The present narrative review aims to introduce the details of piRNA biogenesis and function and then detail the various discoveries as a timeline of events. The review aims to summarize the emerging roles of a relatively new group of sncRNAs, piRNAs, and their interacting PIWI family proteins in lung cancer. Finally, a correlation between the hallmarks of cancer and the piRNA/PIWI family of proteins has been attempted.

## Search methodology

We used PubMed (MEDLINE inclusive), Google Scholar, Web of Science, and Scopus as principal online databases. To explore the literature related to PIWI RNA, the keyword “PIWI RNA” was used. To elaborate on its role in cancer the keywords “PIWI RNA” and “cancer” were used. Keywords “PIWI RNA” and “lung cancer” were used to narrow the specification to lung neoplasms. The genesis and logic for separation of dates to demark “PIWI RNA” and “cancer” and “PIWI RNA” and “lung cancer” as detailed in **Table 1** was lung cancer until end 2021 and other cancers until 2019. This delimited the information and references related to PIWI RNA and cancers other than lung cancer to 2019. Preference was given to citation of references published in the past 5 years. The reference lists of identified articles were further examined for relevant publications. We have arranged our gathered information using the subtitles indicated in the review and further cross-verified each subheading content with appropriate keywords. We have assembled information for readers for ready reference and have added our personal comments summarizing information acquired along with an attempt to collate piRNA with the basics of cancer biology.

### Biogenesis of piRNA

The pathway for piRNA generation is rather complicated and largely uncharted. However, studies conducted so far have confirmed 2 distinct pathways operate in the case of germline and gonadal somatic cells of *Drosophila*. Precursors of piRNAs are mainly transcribed from gene clusters *flamenco* and *traffic jam*. *Flamenco* is one of the major clusters for piRNA biogenesis in the somatic support cells of the *Drosophila* ovary and produces piRNA precursors. The cluster resides in the perichromatin region of the X chromosome in *Drosophila.* An approximately 180 kb stretch of the cluster transcribes into nascent piRNAs, which typically span about 150 kb. The transcripts are generated from a single strand of the DNA by unidirectional transcription orientation in the antisense direction. Partial or an entire loss of *flamenco* in *Drosophila* leads to malfunctioning in transposable element (TE) management [66, 67]. A similar, piRNA-producing locus in chromosome 2 in *Drosophila* is called *traffic jam*, whose primary function was identified as a crucial factor in gonadal morphogenesis in these flies. Loss of *traffic jam* leads to blockade in the differentiation of somatic cells into germ cells and ultimately the formation of follicular cells in *Drosophila* ovaries [68]. Although these 2 clusters are the widely studied and important sources of piRNA, other sources such as intergenic regions and transposons are also noted [69]. In these piRNA regions in the DNA, trimethylated lysine 9 of histone 3 (H3K9Me3) marks are abundant and contribute to piRNA expression [70]. In germ cells, piRNA clusters, to be transcribed, require a protein complex consisting of Rhino, Deadlock, and Cutoff (RDC) proteins [71]. TREX is another complex needed for the dual stranded cluster transcription recruited to the DNA in RDC dependent manner [8]. The length of mature piRNAs varies from 24 to 32 nucleotides [5].

### Zucchini mediated pathway

In the gonadal somatic cells and follicular cells, after transcription, the precursors of piRNAs are transported from the nucleus to the Yb body (containing Piwi, Armitage (Armi), Tudor, Vreteno (Vret), RNA helicase Sister of Yb (SoYb)) in the cytosol [72]. Then premature piRNAs are processed at the 5′ end by a mitochondrial membrane endonuclease called Zucchini (Zuc) [73]. Subsequently, they are loaded onto Piwi by Shutdown and Hsp83 and their 3′ end is trimmed by a slicer enzyme [74]. Piwi has a bias for 5′ U. Aub and Ago3 proteins are not used in this pathway. This processed piRNA is further trimmed by a protein called Nibbler [75]. Ultimately, it is methylated at the 2′ O position by a methyltransferase Hen1 (HENMT1 in mice) [76]. This last step is believed to increase the stability of the piRNA. Processed and mature piRNAs are then transferred back to the nucleus.

### Ping-pong pathway

The secondary amplification of piRNAs occurs in germ cells with the help of AUB and AGO3 proteins via the ping-pong pathway, which also leads to post-transcriptional gene silencing (PTGS). The ping-pong pathway starts with the loading of nascent piRNAs, transcribed from the clusters to Aub. Aub shows a 5′U bias for piRNAs and Ago3 shows a bias for adenine at the 10th position. The 5′ end is loaded on Aub with the help of the Shu and Hsp83 and then trimmed and methylated as described above for the Zuc mediated pathway [74]. piRNAs loaded onto Aub are antisense to specific TE mRNA and they eventually guide Aub to their complementary TE mRNA in the cytosol for targeted destruction. This process also generates the 5′ piRNA precursor. The primary piRNA precursor is loaded onto Ago3 and processed into secondary piRNA precursors. The secondary piRNA precursor is sense to TE and antisense to unprocessed piRNA; accordingly they cleave the newly attached piRNA precursors and continue the loop of transposon silencing and mature piRNA production [77].

### Function

#### Transposon silencing

piRNAs save the germ cells from mutations by TE. The mechanism of piRNA biogenesis in *Drosophila* and many other species is itself a process of degrading TE at the post-transcriptional level. Mutations in Aub and Ago3 lead to an elevated level of transposons in the germ cells [78]. As discussed earlier for the ping-pong pathway, a piRNA targets a transposon with opposite orientation and degrades it into a new piRNA. Together these processes serve to slice TE and amplify piRNA. The transposon-containing regions of the DNA generate piRNAs, which in turn upon maturation, recruit Piwi and other proteins to silence the TE region [79, 80]. Other species, such as mice and zebrafish, also show a similar function for their PIWI family proteins. Knocking down of Piwi orthologs increased the abundance of transposons in these species [81].

#### piRNA mediated epigenetic regulation and transcriptional silencing

Piwi and Aub conduct position effect variegation in which a stretch of euchromatin is converted to heterochromatin to variable extents among cells within the same tissue [82]. Piwi interacts with heterochromatin protein 1a (HP1a) to promote heterochromatin formation [83]. It is shown by studies in *Drosophila* that Piwi helps in loading the H3K9Me3 mark on DNA and converts stretches of DNA into heterochromatin. So, the loss of Piwi makes TE available for pol II [84]. In mice, Mili and Miwi2 (mouse orthologs of PIWI) together promote retrotransposon silencing by CpG DNA methylation in male germ-line cells and piRNA alone can also regulate DNA methylation in these germ cells [81, 85].

#### Reproduction and development

PIWI proteins have stem cell maintaining properties for which their role in germline development and maintenance is well observed. Knockdown studies have shown anomalies in development. For instance, in *Drosophila* both male and female piwi protein-coding gene mutants fail to form primordial germ cells and renew germline stem cells, which leads to sterility [67]. Aub protein-coding gene mutation leads to compromised PTGS and DNA damage accumulation also resulting in sterility [86]. Females with mutant ago3 lay fewer eggs and most of the time are sterile [87]. Similar results were also obtained from studies on mice. PIWI orthologs, Mili, Miwi2, and Miwi mutant mice show defects in PTGS and spermatogenesis [88]. Zebrafish PIWI-like proteins Zili and Ziwi mutations decrease the number of germ cells and increase apoptosis of germ cells respectively [89]. Mutations in other piRNA biogenesis proteins such as Tudor, Vret, and Tej also result in DNA damage and defects in development [74, 79]. piRNA clusters from the chromosomes of *Drosophila* germ cells play a crucial role in maintaining the integrity of the telomeric regions. Loss of piRNAs in germ cells results in decreased levels of HP1a, Rhino and H3K9Me3 association with the telomeric region and disputed nuclear positioning of the telomere [90]. In silkworms and other insects, PIWI has a role in sex determination. Loss of Bmsiwi (insect PIWI) and histone methyltransferase BmAsh2 causes female-to-male sexual reversal [91].

#### Translational regulation by PIWI–piRNA pathway

piRNAs can be formed from the 3′UTR region of many protein-coding genes such as *vas*, *traffic jam*, and *nanos* and control the mRNA turnover and protein level expression of these genes. *Drosophila* flies with piwi mutant flies produce an excess of these gene products, which accumulate in the cell eventually damaging the DNA [70, 92, 93]. Mouse PIWIs interact with eIF4e, which forms a cluster of proteins to control translation [94].

#### Somatic functions

Although very little evidence has been found so far, studies are suggesting a greater somatic function of the PIWI/piRNA pathway. In the early stages of embryogenesis, *Drosophila* piwi is needed for chromatin structure maintenance and cell cycle progression [95]. piwi is also found on chromosomes of the salivary gland that mediate epigenetic regulation [96]. Loss of piwi proteins in *Drosophila* intestinal stem cells impairs the gut regenerative capacity of these flies [97]. In *Aplysia*, piRNA-mediated DNA methylation is needed for neuronal plasticity [98]. In various arthropods, the PIWI-piRNA pathway may perform as an antiviral defense mechanism in mosquitoes and silkworms [99]. In a rat model of diabetes, activities of pancreatic β-cells may be regulated by piRNAs. β-Cells express thousands of piRNAs and their expression changed when rat Piwi proteins were downregulated, resulting in defective insulin secretion [100]. Besides all these major and minor functions of the PIWI–piRNA pathway in various animals, aberrant expression in human cancers has been reported (**Table 1**), which will be discussed in detail in the following sections.

### Role of PIWI–piRNA in lung cancer

#### Cell proliferation

Induced expression of PIWIL2 in A549 cells results in increased cell proliferation by elevated expression of CDK2 and cyclin A, both in vitro and in vivo. Similar results are obtained from H460 cells in vitro. By contrast, RNAi-mediated depletion of the protein results in cell cycle arrest (G2/M) and increased apoptosis [101]. An increase in *PIWIL1* activity induces cell proliferation in A549 cells and decreases in H1299 cell proliferation when knocked down and increases the colony-forming capacity of tumor cells [63, 102].

A positive correlation of cell proliferation with aberrant expression of piRNAs can be postulated. piR-651 is one example of such piRNAs whose expression is altered significantly in many cancers including in lung cancer patient samples and cancer cell lines such as NCIH446 and 95-D [103]. piR-651 maintains the cell population by keeping proapoptotic proteins in check [103]. Inhibition of piR-651 decreases cell proliferation and increases apoptosis in A549 and HCC827 cells. piR-651 negatively regulates proapoptotic proteins while increasing the activities of antiapoptotic proteins [104]. piR-651 helps cyclin D1 and CDK-4 overexpression and upregulates proliferation of transfected A549 cells both in vitro and in vivo [105]. The expression of RASSF1C has been shown to regulate certain piRNA expression and cancer progression. Upregulation of this oncoprotein correlated with overexpression of piR-52200 and underexpression of piR-35127 inpatient samples while piR-34871 and piR-46545 were additionally up- and downregulated respectively in the non-small-cell lung cancer (NSCLC) cell line H1299 along with the previous 2 piRNAs. In vitro, overexpressing underexpressed and knocking down overexpressed piRNAs decreases cell proliferation. Specifically, knock down of piR-52200 in A549 cells, piR-34871 in HT520 cells decreases cell proliferation significantly. By contrast, H1299 responded in most knockdown and overexpression studies. A low level of colony formation was observed in normal lung tissues after manipulation of piRNA expression [106]. piR-55490 acted as an anticancer agent in vitro and in xenograft studies. In lung cancer cell lines such as A549, H460, and H1299, piR-55490 expression was originally suppressed and upon overexpression, these cell lines showed decreased proliferation. It is postulated that piR-55490 binds to mTOR and degrades it decreasing tumor cell proliferation [107]. Two piRNAs overlapping in the 15th chromosome and sharing a common single nucleotide polymorphism, rs11639347, piR-5247, and piR-5671, increase proliferation of A549 cells [108].

#### Stemness maintenance

Human PIWI proteins are now proven to maintain the stemness of certain cell populations when present in testis. Hiwi inhibition resulted in the loss of ALDH-1 (cancer cell marker) positive cells and decreased tumor mass in immunocompromised mice when injecting SSC^lo^ Alde^br^ stem cells isolated from an SPC-A1 cell line [109]. Overexpression of RASSF1C promotes CD133^+^ (stem cell marker) A549 cell tumor sphere formation. RASSF1C induces PIWIL1 expression, which maintains stem cell properties and regulates the wnt/β-catenin pathway. Coexpression of RASSF1C and IGFBP-5 reduces PIWIL1 expression [110].

#### Metastasis

The interplay between PIWIs and piRNAs aids more than one hallmark of cancer. Inhibition of piR-651 decreases migration of highly invasive cell lines 95-D, A549, and HCC827 cells [106, 107]. Inhibition of PIWIL1 interferes with metastatic activity in H1299 cells, while increased expression induces A549 cell migration [102].

#### Overall survival

Human case study databases like The Cancer Genome Atlas (TCGA) show a positive correlation between PIWIL1 expression and poor overall survival of patients [102]. Patient samples show similar results, increased PIWIL1 correlating to shorter time to relapse (TTR) and shorter overall survival. Whereas, decreased PIWIL4 correlated with shorter TTR and less overall survival [102]. Patients with higher piR-55490 expression have longer overall survival [107].

#### piRNA-like short noncoding RNA

Studies in NSCLC and lung squamous cell carcinoma (LSCC) have revealed another class of sncRNAs, the piRNA-Like RNAs (piR-Ls). These RNAs have similar as well as distinguishing features to piRNAs. Two variants have been discovered to date, piR-L-138 and piR-L-163, and both are similar to piRNAs in length. However, 2 major differences are that, unlike piRNAs, they are expressed in adult tissues, and they bind directly to phosphorylated protein targets (p-proteins) to regulate their functional efficacy. Therefore, they are designated as protein functional effector sncRNAs (pfeRNAs). piR-L-138 expression in LSCC increases after cisplatin-based chemotherapy, which eventually leads to chemoresistance by the tumor cells. By contrast, targeting piR-L-138 in LSCC cell lines such as H157 and SKMES-1 increases apoptosis. piR-L-138 regulates p60MDM2 to control cell proliferation [111, 58]. Another study showed piR-L-163 binds to Ezrin, Radixin, and Moesin (p-ERM), which in turn increases the binding capacity of p-ERM to EBP50 and F acting. Blocking piR-L-163 induces cell growth and invasion revealing it as a negative regulator of tumor progression [112].

#### piRNA biomarker and chemoresistance

Cancer prognosis is related to 2 important facets namely early detection and delayed chemoresistance. In a study with 20 pairs of malignant and nonmalignant tissues, piR-hsa-211106 was downregulated in all malignant tissues as it prevented metastasis and induced apoptosis in lung cancer cells [61]. This piRNA interacts with pyruvate carboxylase, which prevents cisplatin resistivity in lung cancer cells [61].

Lin et al. [60] and Li et al. [62] demonstrated that piRNAs can also be used for diagnosis of lung cancer. Sputum from 32 lung cancer patients was used as a source of epithelial cells from the bronchus and cRNA was profiled [60]. Lung cancer patients had upregulated piR-004987 and piR-020809 expression and downregulated expression of piR-023338 and piR-011186 [60]. Li et al. [62] examined 19 lung tissues from lung cancer patients and compared their piRNA profile with noncancerous lung tissues (from different sites in the same patients). They found 10 piRNAs unregulated in cancerous tissues compared with noncancerous tissue samples (see **Table 1**). Of these, 2 exosomal piRNAs, namely piR-hsa-26925 and piR-hsa-5444, were found in patient sera. Exosomes are double-layered lipid extracellular vesicles containing macromolecules like nucleic acids (in this case piRNA) used for cell-to-cell communication. Thus, such exosomes identifiable from sera can act as diagnostic markers for lung cancer.

## Conclusion

**Figure 2:**
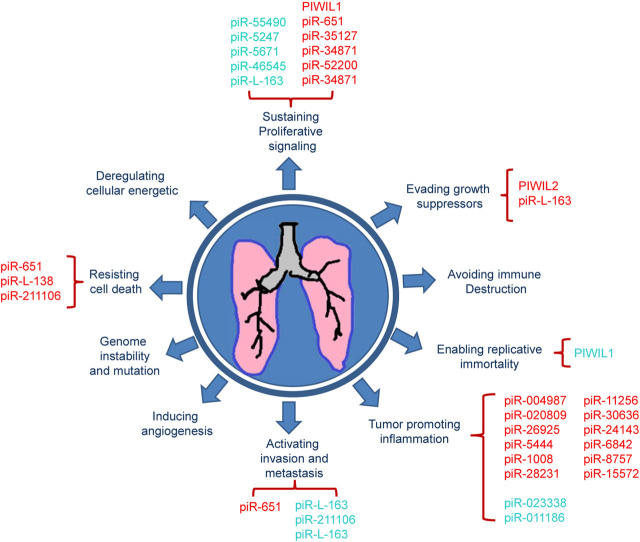
Representation of how the present findings for PIWI proteins or piRNA integrate with the emerging hallmarks of cancer in the case of lung cancer. Turquoise represents positively regulated with the hallmark, while red represents negatively regulated with the hallmark. piRNA, PIWI-interacting RNA; P-element induced wimpy testis protein.

Accumulated data supports the importance of piRNAs in lung and other cancers. Presently we have emphasized lung cancer as it still reigns among all types of cancers in terms of the highest mortality in cancer-related deaths worldwide [1]. Lung cancer management, like any other cancer, revolves around both diagnoses and treatment. For both these factors, detailed molecular understanding of the disease is necessary for an effective outcome. piRNA contributes to cardinal features of cancer development, namely, cell proliferation, stemness maintenance, and metastasis; thus, also reflecting overall survival. Better understanding and clinical interpretation of these noncoding RNAs will not only aid in the understanding of the molecular perturbations, but may also provide insight into the selection of treatment modalities. More detailed screening and identification of anomalous expression of piRNAs may not only help in diagnosis, but also predict the prognosis of the disease. To collate the basics of cancer biology with the advances with knowledge of PIWI proteins or piRNAs, we merged our gathered information with the “Emerging hallmarks and enabling characteristics” as delineated by Hanahan and Weinberg [113, 105]. A diagrammatic representation of how the present finding of PIWI proteins or piRNA integrates with the hallmarks of cancer is depicted in **Figure 2**. Those PIWI proteins or piRNA that are positively regulated with the hallmarks may serve as a target for lung cancer therapy or as diagnostic or prognostic markers. By contrast, negatively regulated PIWI proteins or piRNA may serve as therapeutic options.
